# On Mounting Preparations under Thin Glass

**Published:** 1845-09

**Authors:** J. W. Griffith


*On Mounting Preparations under Thin Glass.* By Dr. J. W. Griffith.—The preparations which I have found answer permanently are—" 1st, A solution of Canada balsam in ether or oil of turpentine, evaporated to just such a consistence as is sufficient to allow of its being applied with a camel-hair pencil. 2d, A mixture of gold size and white lead ; this used as the ordinary gold size and lamp black has remained permanent ; a little red lead mixed in with it, makes it dry quicker and harder. 3d, A mixture of red lead and gold size used immediately, dries very rapidly, and becomes very hard. 4th, A mixture of fine lamp black and white hard varnish laid on immediately forms a very good compound."—*Lond. and Ed. Month. Journ. of Med. Sci., from Tulk and Henfrey's Anatomical Manipulation.*

